# From the immune system to mood disorders especially induced by *Toxoplasma gondii*: CD4^+^ T cell as a bridge

**DOI:** 10.3389/fcimb.2023.1078984

**Published:** 2023-04-03

**Authors:** Qing Wang, Yue Zhong, Nannan Chen, Jinling Chen

**Affiliations:** Department of Pathogen Biology, School of Medicine, Nantong University, Nantong, Jiangsu, China

**Keywords:** mood disorders, *Toxoplasma gondii*, CD4^+^ T cell, regulatory T cells, Th17

## Abstract

*Toxoplasma gondii* (*T. gondii*), a ubiquitous and obligatory intracellular protozoa, not only alters peripheral immune status, but crosses the blood-brain barrier to trigger brain parenchymal injury and central neuroinflammation to establish latent cerebral infection in humans and other vertebrates. Recent findings underscore the strong correlation between alterations in the peripheral and central immune environment and mood disorders. Th17 and Th1 cells are important pro-inflammatory cells that can drive the pathology of mood disorders by promoting neuroinflammation. As opposed to Th17 and Th1, regulatory T cells have inhibitory inflammatory and neuroprotective functions that can ameliorate mood disorders. *T. gondii* induces neuroinflammation, which can be mediated by CD4^+^ T cells (such as Tregs, Th17, Th1, and Th2). Though the pathophysiology and treatment of mood disorder have been currently studied, emerging evidence points to unique role of CD4^+^ T cells in mood disorder, especially those caused by *T. gondii* infection. In this review, we explore some recent studies that extend our understanding of the relationship between mood disorders and *T. gondii*.

## Introduction

Mood disorders are a group of global common mental diseases, recognized by the World Health Organization as one of the major causes of disability all over the world, seriously affecting health and life expectancy, and often imposing a significant economic burden on society (Moussavi et al., 2007; McCarron et al., 2016), which mainly includes major depression disorder (MDD) and bipolar disorder (BD) (Thase and Denko, 2008; Whiteford et al., 2013). MDD has a lifetime prevalence of 2%-15% and BD has a lifetime prevalence of about 2%, both of which contribute to increased mortality (Grande et al., 2016; Park and Zarate, 2019). Patients with mood disorders suffer from cognitive decline, anxiety and suicidal thoughts with varying degrees of behavioral changes (Rakofsky and Rapaport, 2018). Little is known about the pathophysiology of mood disorders, and relevant mechanistic hypotheses are mainly derived from neuroinflammation, genetics, neurotransmitters, neurotrophic factors, and stress axis. However, there is growing evidence to support the involvement of immunoinflammatory pathways in the mechanisms of mood disorders (Sayana et al., 2017). Mood disorders are closely related to the immune system, and changes in the peripheral and central immune environment have been shown to be involved in the pathophysiological process of mood disorders (Dantzer et al., 2008). Patients with mood disorders have been observed to have inflammatory signals and immune cells transmitted from the periphery to the brain, as well as activated microglia (Miller and Raison, 2016). Studies have found the increased levels of CD4^+^ T cells in peripheral blood of MDD patients or groups with anxiety and depression symptoms (Beurel et al., 2020). The main subtypes of CD4^+^ T cells are regulatory T cells (Tregs), T-helper 1 (Th1), T-helper 2 (Th2), and T-helper 17 (Th17). Several experiments have shown that in the peripheral blood of patients with mood disorders, Tregs are reduced (Bauer and Teixeira, 2021), Th17 cells and their marker cytokine interleukin (IL)-17 are increased (Chen et al., 2011; Davami et al., 2016), accompanied by an increase in Th1 cells, Th1/Th2 ratio and Th1/Th2 cytokine levels with pathological imbalance (Huang and Lee, 2007; Beurel et al., 2020). A new study suggests that metabolic disturbance of CD4^+^ T cells is a key factor in chronic stress anxiety, a high-risk symptom of MDD (Jacobson and Newman, 2017; Fan et al., 2019).

*Toxoplasma gondii* (*T. gondii*), an obligate intracellular parasite from the phylum Apicomplexa with a complex, multistage life cycle, infects approximately one-third of the global population (White et al., 2014; Lourido, 2019). Felines are the definitive hosts of *T. gondii*, and humans possibly acquire infection by eating undercooked meat containing cysts, food or water contaminated with oocysts, and mother-to-child transmission (Halonen and Weiss, 2013). Depending on the host immune function, toxoplasmosis can manifest as asymptomatic infection, encephalitis, chorioretinitis, multi-organ involvement, or genotypic infection (Galli et al., 2019). *T. gondii* is capable of being present in warm-blooded animal tissues, especially muscle and brain, in the form of cysts, but it can only complete its life cycle by reproducing sexually in the feline body. So to complete its life cycle progression, this parasite evolved to influence host behavior, such as changing from an aversion to cat odor to a preference and increased mobility, making its ingestion by cats more likely. Indeed, *T. gondii* is neurotropic and may be associated with structural abnormalities of the brain, capable of forming cysts in the brain parenchyma and remaining in the host brain for life, infecting microglia, astrocytes, neurons and stimulating the production of multiple cytokines that affect host behavior and cognitive function (Coryell et al., 2020; Erickson et al., 2021; Gale et al., 2021).

In recent years, there has been an increasing interest in parasites affecting human behavior and mental disorders. The main parasites affecting central nervous system (CNS) include *T. gondii*, *Trypanosoma brucei*, *Entamoeba histolytica*, *Plasmodium falciparum*, and *Schistosoma japonicum* (Gondim et al., 2019). These parasites are all capable of establishing infections in the brain and are associated with cognitive and behavioral changes and psychiatric disorders, and although the causal mechanisms between them have not been elucidated, the association is plausible (**Table 1**). In particular, *T. gondii* is the most common neurotropic protozoan in humans, and an increasing number of epidemiological studies have highlighted the strong association of *T. gondii* infection with mood disorders and its involvement in their pathogenesis (Suvisaari et al., 2017; Galli et al., 2019; Yalin Sapmaz et al., 2019; Lin et al., 2020). For instance, *T. gondii* IgG antibody titers were also positively correlated with anxiety and depression in pregnant women (Groer et al., 2011; Nourollahpour Shiadeh et al., 2016). The prevalence of *T. gondii* infection detected by immunohistochemistry is around 8.0% (7/87) in the prefrontal cortex and amygdala in suicide victims, and *T. gondii* infection was highly associated with a history of MDD in suicides (Alvarado-Esquivel et al., 2021).

**Table 1:** Parasites affecting CNS and associated with mood disorders.

Parasites	Is it neurotro-pic?	target tissue	Related Psychiatric Symptoms	Types of psychiatric disorders	Possible causes or mechanisms	References
*Toxoplasma gondii*	Yes	Brain; Intestine; Lungs; Eyes; Heart;	Suicidal behavior; cognitive impairment; impaired social skills; anxiety and depression-like behavior;	Schizophrenia; BD; anxiety disorders; MDD;	Brain cyst load; neuroinflammation; structural and functional pathological alterations of neurons; synaptic and neurotransmitter abnormalities; tryptophan pathways;	(Torrey and Yolken, 2003; Groer et al., 2011; Robert-Gangneux and Darde, 2012; Schluter and Barragan, 2019; Tyebji et al., 2019; Yalin Sapmaz et al., 2019; Xiao, 2020; Postolache et al., 2021; Cossu et al., 2022)
*Trypanosoma brucei*	No	Brain;	Impaired motor and exploratory skills; anxiety-like behavior;	?	Central nervous system lesions; meningoencephalitis; increased pro-inflammatory cytokines;	(Stich et al., 2002; Darsaud et al., 2003; Saric et al., 2010; Adebiyi et al., 2021)
*Entamoeba histolytica*	No	Intestine; Liver; Lung; Brain; Skin;	Altered mental status;	?	Meningoencephalitis; destruction of normal brain structures;	(van Hal et al., 2007; Ong et al., 2017; Berger, 2022)
*Plasmodium falciparum*	No	Brain; Kidneys; Lungs; Spleen; Cerebellum;	Behavior change; cognitive impairment;	Mania; depression; anxiety;	Neurodevelopmental disorders; neuroinflammation; neuronal death;	(Newton, 2005; de Miranda et al., 2011; Moxon et al., 2020; Akide Ndunge et al., 2022,; Trivedi and Chakravarty, 2022)
*Schistosoma japonicum*	No	Intestine; Liver; Skin; Lungs; Adrenal glands; Skeletal muscle; Spinal cord; Brain;	Mobility and cognitive impairment;	Depression; anxiety;	Granuloma formation in the brain; neuroinflammation;	(Jia et al., 2007; Jia et al., 2011; Ross et al., 2012; Tan et al., 2019)
*Taenia solium*	No	Brain; Eyes; Spinal cord; Skeletal muscle; Skin; Heart;	Cognitive impairment; memory deficits;	Depression;	Cyst formation in the brain; inflammation of the brain; neuronal damage;	(Del Brutto et al., 2001; Ciampi de Andrade et al., 2010; Garcia et al., 2020; Garcia-Martinez et al., 2022)
*Trichinella spiralis*	No	Muscle; Stomach; Intestines; Lungs; Heart; Brain;	Behavioral disorders; cognitive disorders; altered mental status;	Depression; anxiety;	Inflammation of the brain; neurological impairment;	(Clausen et al., 1996; Taratuto and Venturiello, 1997; Rosca et al., 2021)

However, some studies have not found a link between *T. gondii* and mood disorders (Galli et al., 2019; Gao et al., 2019; Nayeri Chegeni et al., 2019). The contradictory findings may be due to differences in sample size, as samples for such studies are not easy to obtain, geographical differences between samples, life span of infection, differences in *T. gondii* genotypes around the world, factors such as life circumstances, economic status, life stress, and MDD or BD typing can alter the association between *T. gondii* infection and mood disorders. Moreover, differences in the type and dose of *T. gondii* strains used to induce behavioral changes during the construction of animal models, as well as host species and sex, can lead to inconsistent study results (Worth et al., 2014). Although the causal relationship between *T. gondii* infection and mood disorders is not yet clear, research on them is full of implications, and indeed, studies on the relationship between *T. gondii* and MDD or BD have been increasing in the last decade.

**Figure 1:**
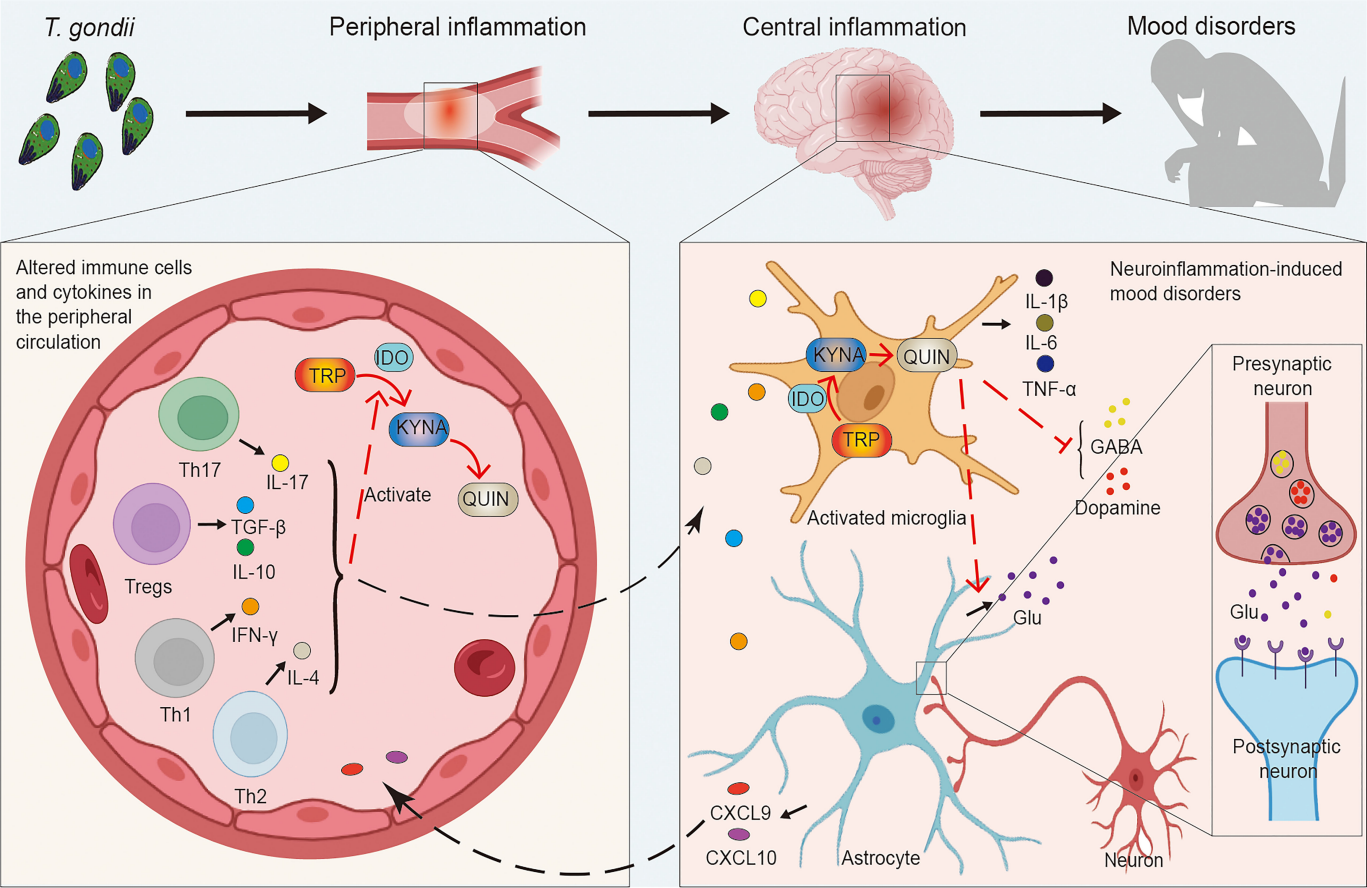
Links between *Toxoplasma gondii*-induced peripheral and central inflammation and mood disorders. *T. gondii* infection induces an imbalance in a series of immune cells (e.g. Tregs, Th1, Th2, Th17, etc.) as well as cytokines (e.g. IL-4, IL-10, IL-17, IFN-γ, TGF-β, etc.), causing peripheral inflammation. Inflammatory cytokines activate IDO to promote the decomposition of TRP into KYNA and QUIN, while peripheral inflammation can influence the CNS, which induces central neuroinflammation and affects neurons as well as neural circuits. Microglia are activated to release inflammatory factors (e.g. IL-1β, IL-6, TNF-α, etc.) and promote the TRP pathway. Simultaneously, QUIN activates increased Glu release from astrocytes, leading to the increased levels of intra- and extra-synaptic Glu, while the production and release of neurotransmitter dopamine and GABA are inhibited as well. This complex series of responses induces the development of mood disorders. CNS, central nervous system; GABA, gamma-aminobutyric acid; Glu, glutamate; IDO, indoleamine 2, 3-dioxygenase; IFN, interferon; IL, interleukin; KYNA, kynurenine; QUIN, quinolinic acid; TGF, transforming growth factor; Th, T-helper cells; TNF, tumor necrosis factor; Tregs, regulatory T cells; TRP, tryptophan; *T. gondii*, *Toxoplasma gondii*.

This article focuses on the positive association between *T. gondii* and mood disorders and analyzes the possible mechanisms of this association, with the immune system serving as a possible bridge between the two *T. gondii* infection alters the peripheral and central immune status, with an increase in peripheral CD4^+^ T cells and their recruitment into the brain parenchyma, accompanied by the development and transfer of inflammation (Ploix et al., 2011; Severance et al., 2016), or directly into the CNS to promote microglia activation (Courret et al., 2006; Matta et al., 2021) (**Figure 1**). *T. gondii* is involved in the pathological development of neuropsychiatric disorders by communicating with the CNS through the immune system as a bridge. Interestingly, this mechanism is similar to the microbial “gut-brain axis”, in that gut microbes have been shown to communicate with the brain, including through the immune system, in neuropsychiatric disorders such as anxiety, schizophrenia, Parkinson’s disease and Alzheimer’s disease (AD) (Cryan et al., 2019).

## *T. gondii* life cycle and strain types

The life cycle of *T. gondii* has three main distinct developmental stages: oocysts containing sporozoites, tachyzoites, and tissue cysts containing bradyzoites. Oocysts are the product of sexual reproduction of *T. gondii*, which occurs only in the epithelial cells of the intestine of the definitive host (felines). In stark contrast to oocysts, tachyzoites and bradyzoites are the other two asexual reproductive stages in the cycle of *T. gondii.* When intermediate hosts (warm-blooded animals) ingest sporulated oocysts in feline feces, the sporozoites in the oocysts are released in the small intestine of the intermediate hosts and infect the epithelial cells, at which point they transform into tachyzoites, which then form parasitic vesicles and enter a rapid division phase. The tachyzoites are the most destructive pathogenic stage in the life cycle of *T. gondii*, dividing and attacking all tissues of the host until they are controlled by the host’s immune system. The tachyzoites then divide into bradyzoites, which divide slowly and remain dormant, mainly in the brain, bones and heart and form tissue cysts to evade the host’s immune response, thus causing persistent infection (Blader et al., 2015; Elsheikha et al., 2021). Humans acquires infection mainly *via* ingesting food or water, which is contaminated with oocysts or tissue cysts of *T. gondii*. Benefit from the development of the *T. gondii*-Cre system, which is capable of identifying host-parasite interactions and imaging infected cells, it was found that *T. gondii* invades parenchymal cells such as astrocytes and neurons after CNS invasion, but it prefers to infect neurons and form cysts in neuronal synapses (Cabral et al., 2016).

The severity of disease caused by *T. gondii* infection also varies greatly, and this is highly dependent on the type of strain of *T. gondii* as well as the host. It is generally accepted that there are three main clonal lineages of *T. gondii*, types I, II and III. This typology is based on a large number of studies of *T. gondii* genotypes in Europe and North America, but a different lineage from these three types has since been identified in Brazil. Genetic analysis of *T. gondii* in Asia also identified ToxoDB#9 (Chinese 1), which is distributed in many provinces of China, and is considered to be an important clonal lineage of *T. gondii* alongside types I-III, which are also common in Asia (Pena et al., 2008; Chaichan et al., 2017). Different strains of *T. gondii* are not equally virulent, with Type I being very virulent and 100% lethal (LD100) in a single injection of *T. gondii* in the house mouse (Mus musculus -M.m.- domesticus), Type II being a low virulence strain (LD50>103) and Type III being even less virulent (LD50>105) (Fukumoto et al., 2020). This virulence of different strains is also reflected in humans, for example, human ocular toxoplasmosis is not associated with types II and III, but is associated with type I (Grigg et al., 2001). Intriguingly, the infectivity of *T. gondii* is species-dependent. *T. gondii* strains endemic in a given region have extremely low virulence against native mice. For example, types II and III endemic in Europe and North America do not kill the major European and North American mouse subspecies M. m. - domesticus, whereas type I endemic in North and East Asia is extremely virulent against M. m. - domesticus but less virulent against the major local mouse subspecies M. m. castaneus and M. m. musculus. Some animals living in the absence of felines or in higher habitats away from oocysts (e.g. wallabies and new world monkeys) are highly susceptible to *T. gondii*, whereas humans and some farm animals (e.g. cattle, sheep, pigs) are more resistant to *T. gondii* (Mukhopadhyay et al., 2020).

Hosts such as humans and mice can sense *T. gondii* infection through pattern recognition receptors such as toll-like receptors (TLRs). After *T. gondii* has invaded the organism and is sensed, three major signaling pathways are activated: (i) nuclear factor κB (NF-κB), (ii) mitogen-activated protein kinases (MAPKs), and (iii) IFN regulatory factors (IRFs). Activation of these pathways induces the production of IL-12, tumor necrosis factor (TNF)-α and interferon (IFN) and the recruitment of immune cells such as monocytes, neutrophils and dendritic cells (DC) to the site of infection. IL-12 stimulates the production of IFN-γ by natural killer (NK) cells and T cells (CD4^+^ and CD8^+^), which are important mediators of parasite eradication (Dupont et al., 2012; Mukhopadhyay et al., 2020). CD4^+^ and CD8^+^ T cells are also activated during the course of infection. It has been reported that IFN-γ-mediated macrophage activation and CD8^+^ T cell-mediated cytotoxicity act in the acute and chronic phases of *T. gondii* infection, respectively, and that CD8^+^ T cells can induce the elimination of *T. gondii* cysts through perforin-mediated cytotoxicity against chronic infection with *T. gondii*. The CD4^+^ T cell population has an important helper function in CD8^+^ T cells, *via* increasing numbers and enhancing function of CD8^+^ T cells. *T. gondii* infection induces a strong CD4^+^ T cell response, which is the main source of IFN-γ during infection (Suzuki et al., 2010; Khan et al., 2019). IFN-γ strongly stimulates cellular autonomic immune responses in response to infection, induces cellular expression of GTPase, carbon monoxide synthase and indoleamine 2, 3-dioxygenase (IDO) expression, inhibits *T. gondii* growth and mediates direct killing (Sasai et al., 2018).

## *T. gondii* marches to the brain

**Figure 2:**
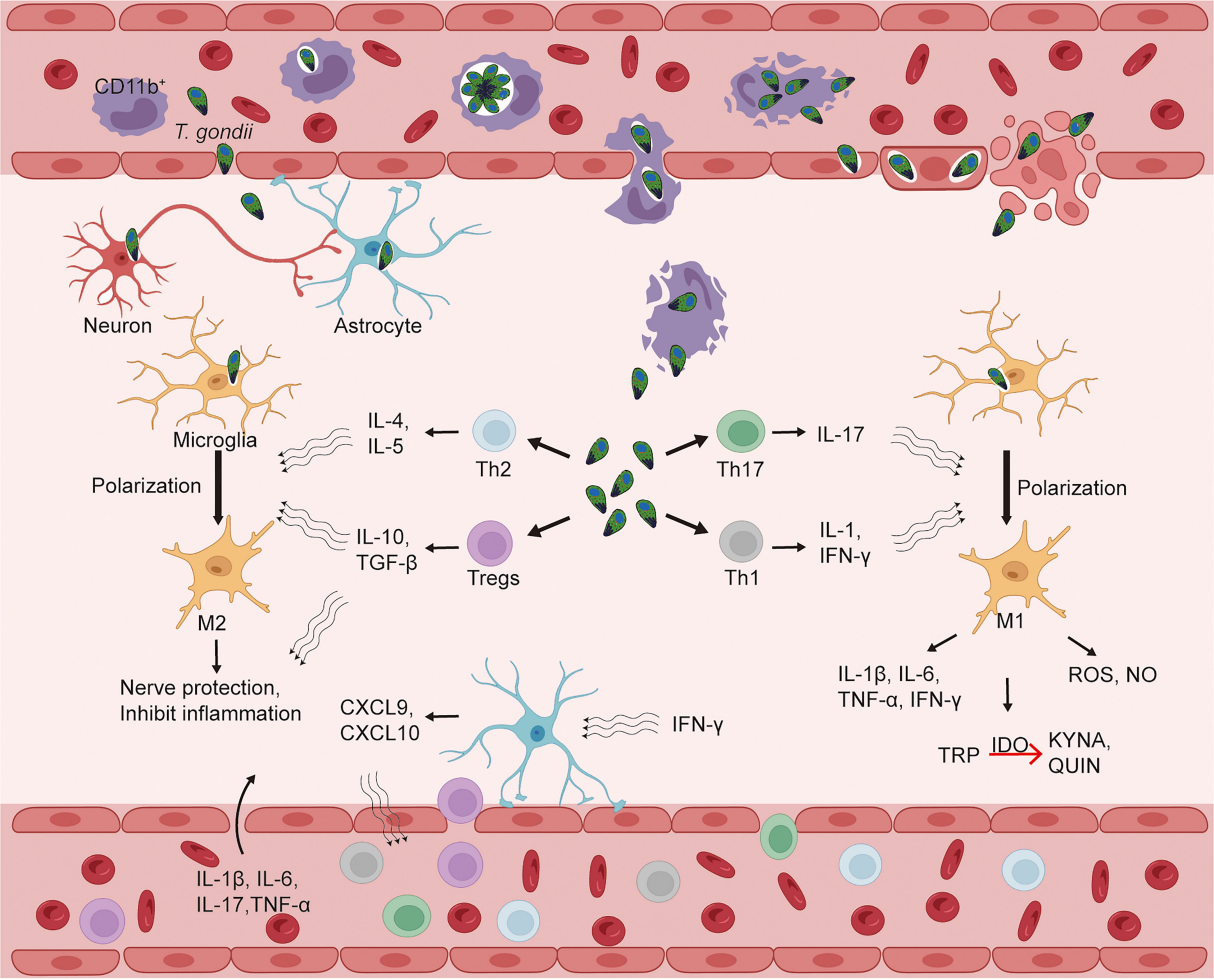
*Toxoplasma gondii* infects the central nervous system and CD4^+^ T cells enter the brain parenchyma, causing alterations in the immune status of the brain. The blood-brain barrier (BBB) is an important barrier to protect the central nervous system (CNS) from external aggression. *T. gondii* can enter the bloodstream after infection and then break through the BBB to the CNS, which may be achieved by three mechanisms: a “Trojan horse” mechanism that relies on CD11b^+^ monocytes; BBB endothelial cell lysis; and the BBB paracellular pathway. After entering the CNS, *T. gondii* invades a variety of parenchymal cells, such as microglia, astrocytes, and neurons. *T. gondii* infection can cause alterations in the levels of CD4^+^ T cells (Tregs, Th1, Th2, Th17) and some cytokines in peripheral blood, while IFN-γ in CNS can activate astrocytes to produce chemokines (CXCL9, CXCL10) to induce CD4^+^ T cells to enter CNS *via* BBB in response to *T. gondii* infection. In the brain parenchyma, cytokines released by Tregs, Th1, Th2, and Th17 can polarize microglia into M1 or M2 types, which can exert pro-inflammatory/neurotoxic or anti-inflammatory/neuroprotective effects, respectively. CXCL, CXC ligand; IDO, indoleamine 2, 3-dioxygenase; IFN, interferon; IL, interleukin; KYNA, kynurenine; QUIN, quinolinic acid; TGF, transforming growth factor; Th, T-helper cells; Tregs, regulatory T cells; TRP, tryptophan; *T. gondii*, *Toxoplasma gondii*.

*T. gondii* can metastasize to a variety of tissues, including the eyes, heart, liver, lungs, lymph nodes and muscles. More importantly, it can enter the brain, invade the CNS and cause encephalitis, establishing a persistent chronic infection in nerve and other brain cells (Elsheikha et al., 2021). After *T. gondii* infects the host, it can invade CD11b^+^ monocytes, which then carry *T. gondii* and spread to distant tissues along with the blood circulation. *T. gondii* can rely on this “Trojan horse” pathway to invade and colonize the brain parenchyma through the blood-brain barrier (BBB) (Courret et al., 2006). Endothelial cells (ECs) can also act as a bridge for *T. gondii* to invade the CNS. Free *T. gondii* in the peripheral circulation and released *T. gondii* from positive monocytes can invade cerebral vascular ECs, replicate in ECs, and then dissolve ECs to damage the BBB, and finally enter the CNS (Konradt et al., 2016). In parallel, *T. gondii* can also use the paracellular pathway to enter the brain parenchyma directly through the endothelial cell space of BBB. In conclusion, there are three possible ways that *T. gondii* invades the CNS: CD11b^+^ monocyte-dependent “Trojan horse” mechanism; BBB endothelial cell lysis; BBB paracellular pathway (Matta et al., 2021) (**Figure 2**). Further analysis of the entry of *T. gondii* into the brain parenchyma revealed that in humans and mice, initial infection with *T. gondii* in the brain parenchyma was mild or non-existent (Ross et al., 2022). The minimal number of *T. gondii* in the brain parenchyma compared to initial infections in other organ parenchyma (spleen, liver, lung) facilitates the avoidance of a strong immune response to remove *T. gondii*, allowing for progressive development and persistent infection in the CNS. In the late stages of infection the brain endothelium becomes infiltrated with leukocytes and there is a marked inflammatory response which promotes the passage of *T. gondii* through the BBB. In addition, *T. gondii* can facilitate crossing the BBB through protein tyrosine kinase 2 (PTK2), which regulates cell adhesion signaling, and integrin-dependent enhancement of host cell migration. Of course, the complex mechanisms of how *T. gondii* enters the CNS have not been extensively studied, but this process must be clarified if the problem of cerebral toxoplasmosis is to be solved.

After reaching the CNS, *T. gondii* forms cysts mainly in neurons and establishes persistent or latent infection. Significantly, recent studies have found that IFN-γ stimulates neurons and upregulates IFN-γ-responsive related genes and proteins [e.g., GTPase system (IRG)] *in vivo* and *in vitro*, killing intracellular *T. gondii* in neurons *via* IRG and mediating *T. gondii* resistance in neurons. The data suggest that IFN-γ reduces neuronal infection in both mouse and human neurons, but this clearance efficiency is strain type dependent, for example, RH strains are IRG resistant and cannot be cleared (Chandrasekaran et al., 2022). This important IFN-γ-dependent mechanism of *T. gondii* clearance may be the focus of research on cerebral toxoplasmosis. *T. gondii* can also severely infect microglia, and activated microglia play a central role in neuroinflammation (Gonzalez and Pacheco, 2014; Matta et al., 2021). Microglia are the primary cells of immune defense after pathogens have passed through the BBB, and activated microglia can produce pro-inflammatory cytokines (like IL-6, IL-1, and IFN-γ) (Forrester et al., 2018). In a mouse model of *T. gondii* infection, 30% of microglia were found to be invaded (da Silva and Langoni, 2009). Brain-resident cells (most likely microglia) can produce IFN-γ and thereby can inhibit *T. gondii* replication in the brain, while activating astrocyte cells to produce chemokines CXC ligand (CXCL) 9 and CXCL10 to recruit immune cells (such as CD4^+^ T cells) from the peripheral blood to the CNS (Schluter and Barragan, 2019; Suzuki, 2020). Pharmacological studies have found that arctigenin (AG) and ginsenoside Rh2 (GRh2) can ameliorate host or host offspring MDD caused by *T. gondii* infection, mainly because AG and GRh2 can inhibit microglia activation and neuroinflammation *via* the TLR4/NF-κB, TNF receptor 1 (TNFR1)/NF-κB signaling pathways and high mobility group box 1 (HMGB1)/TLR4/NF-κB signaling pathway, respectively (Cheng et al., 2020; Xu et al., 2022).

*T. gondii* may contribute to the onset and development of psychiatric symptoms by altering brain structure and function, such as cytokine-induced neuroinflammation; abnormalities in brain parenchyma and alterations in neuroplasticity; and imbalances in neurotransmitters such as dopamine and glutamate (Galli et al., 2019). Data from experiments in mice suggest that *T. gondii* infection can promote inflammation in the brain. In chronically infected mice, distinct areas of inflammation appear in the soft meninges, hippocampus and perihippocampal vessels, and hippocampal abnormalities are associated with many human neurological disorders, including AD, depression and schizophrenia. Both the degree of *T. gondii* cysts and the level of inflammatory factors have been shown to be highly correlated with behavioral changes in mice (Hermes et al., 2008; Boillat et al., 2020). There is now a consensus that there is an association between *T. gondii* infection and Psychiatric Disorders. Individuals with chronic *T. gondii* infection are more likely to develop psychiatric disorders such as bipolar disorder, MDD, anxiety, and schizophrenia (Nolan, 2019; Tyebji et al., 2019). We have described above the mechanisms by which *T. gondii* enters the brain and the immune changes induced in the brain, which set the stage for a specific mechanism of association between *T. gondii* infection and psychiatric disorders. The immune activation and formation of an inflammatory environment in the brain caused by *T. gondii* infection is an important basis for the pathology of behavioral changes and mood disorders, and further avenues of research include whether the negative effects of *T. gondii* infection on the peripheral and central immune systems make individuals with *T. gondii* infection more susceptible to mood disorders. This may drive the development of drugs to prevent infection and alleviate and ameliorate psychiatric disorders brought on by *T. gondii*.

## Inflammation and mood disorders

*T. gondii* invasion of organisms not only induces innate and adaptive immune responses, but also alters the immune state in the brain, where large numbers of immune cells and inflammatory factors accumulate in the CNS to form a pro-inflammatory state, and this inflammatory state in the brain is associated with the pathogenesis of mood disorders. *T. gondii* infection has been found to cause immune dysregulation, activating peripheral and central inflammatory responses, and induce an imbalance of pro-inflammatory factors (such as IL-6, IFN-γ) and anti-inflammatory factors (such as IL-10) (Del Grande et al., 2017). The CNS inflammatory response can induce the symptoms of mood disorders and participate in the occurrence and development of mood disorders (Martynowicz et al., 2019; Boillat et al., 2020). CD4^+^ T cells are critical for resistance to *T. gondii* infection, and toxoplasmosis often leads to alterations in the number and function of CD4^+^ T cells (Dupont et al., 2012). Studies have demonstrated that *T. gondii* infection inhibits Tregs proliferation, resulting in a decrease in Tregs levels (Gao et al., 2021). However, *T. gondii* triggers a strong Th1 cell response to defend against pathogen infection (Sana et al., 2022), while Th2 cells can suppress the Th1 cell response, so the Th1/Th2 balance plays a vital role during *T. gondii* infection (Xu et al., 2021a). Interestingly, IL-27 inhibits pathological Th17 cell responses during CNS infection by *T. gondii*, preventing excessive inflammatory responses (Stumhofer et al., 2006). In conclusion, *T. gondii* infection leads to chronic CNS infection and neuroinflammation (Klein et al., 2017), and even before *T. gondii* enters the CNS, the peripheral immune system can be activated to produce large numbers of pro-inflammatory cells, leading to neuroinflammation (Tyebji et al., 2019), including altered numbers of CD4^+^ T cells and secretion of cytokine IFN-γ (Aliberti, 2005; Yarovinsky, 2014).

Studies have shown that in patients with MDD and BD, the level of pro-inflammatory factors in peripheral blood will increase, IFN-γ, IL-6, IL-1, and TNF-α involved (Sayana et al., 2017; Fries et al., 2019). Also in a count of 606 MDD patients, an increase in peripheral blood C-reactive protein (CRP) was found, with approximately 47% of patients having high CRP levels (≥ 3.0 mg/L) and roughly 29% of patients having higher CRP levels (≥ 5.0 mg/L) (Rethorst et al., 2014). Interestingly, the use of inflammatory cytokines can induce depression-like behavior in experimental animals and humans, such as long-term acceptance of IFN-α. Up to 30%-50% of treated patients have MDD symptoms (Haroon et al., 2012). Chronic *T. gondii* infection can lead to the development of neuroinflammation, which is also thought to be responsible for behavioral abnormalities (Hermes et al., 2008; Boillat et al., 2020). There is growing evidence that mood disorders and inflammation are intertwined and reinforce each other. Mood disorders tend to facilitate inflammatory responses and exhibit characteristics of inflammation, which in turn promotes mood disorders and other neuropsychiatric disorders (Bauer and Teixeira, 2019).

So how does inflammation affect mood disorders? Cytokines, although relatively large protein molecules (15-25 KD), have been shown to enter the brain and drive CNS inflammation. It can directly or indirectly affect CNS function through BBB penetration, automatic transport of saturated transport molecules, activation of cerebrovascular intimal cells, and binding to receptors on peripheral afferent nerve fibers (Miller et al., 2009). Once cytokines enter the brain, they affect neurotransmitters and neural circuits. Cytokines activate IDO and alter tryptophan (TRP) (a major precursor of serotonin) metabolism, thereby reducing serotonin production and increasing kynurenine acid (KYNA) levels to induce MDD. On the other hand, KYNA can be converted to quinolinic acid (QUIN), which induces astrocyte glutamate (GLU) release and inhibits reuptake, resulting in increased intra- and extra-synaptic GLU levels. Higher GLU levels are associated with increased depressive symptoms. In addition, cytokines have been shown to reduce the release of dopamine, which can lead to decreased motivation and anhedonia, the core symptoms of MDD (Miller and Raison, 2016). The effects of cytokines on neural circuits (basal ganglia, anterior cingulate cortex), neurogenesis, and neuroendocrine functions (such as the hypothalamic-pituitary-adrenergic axis) are systematically described, all of which are highly related to the occurrence and development of mood disorders (Haroon et al., 2012).

Like many infections, the body’s immune cells and cytokines and their induced effector mechanisms control *T. gondii* infection, yet severe infection-induced hyperinflammation can induce the pathology of CNS. There is growing evidence that inflammation can affect the brain, and that the production of inflammatory responses can lead to depressive symptoms and increase the risk of psychiatric disorders such as BD, MDD, and anxiety disorders (Miller, 2020). As a foreign pathogen, *T. gondii* can induce inflammation in the peripheral and CNS (McGovern et al., 2021; Saraav et al., 2021), which may speculate that the inflammatory response is a hub between *T. gondii* and mood disorders, and some inflammatory factors may be the protagonists in this link mechanism. Therefore, it is highly likely that *T. gondii* is involved in the pathogenesis of mood disorders by indirectly affecting peripheral and central immune status and inducing CNS inflammation and this immunological hypothesis may support the pathogenesis of some neuropsychiatric disorders or behavioral abnormalities in which *T. gondii* is involved. We next focus on CD4^+^ T cell numbers and function during *T. gondii* infection and mood disorders.

## Tregs and mood disorders

Tregs are a cell population essential for immune regulation, which modulates pathogen-induced innate and adaptive immune responses. And after *T. gondii* invades the organism, it will cause an immune response, and the number and function of Tregs will also alter (Hall et al., 2012). Tregs were significantly reduced in number and function collapsed in a model of *T. gondii* oral infection (Oldenhove et al., 2009). When *T. gondii* infected pregnant humans or mice, it was also accompanied by a decrease in the number of Tregs (Rezende-Oliveira et al., 2020). Significantly, the immunosuppressive effect of Tregs impedes host immune defenses and increases pathogen susceptibility, so a reduction in Tregs numbers and functional collapse may rescue the organism from the pathological hazards caused by infection. In this section we describe the immunosuppressive and regulatory inflammatory functions and neuroprotective effects of Tregs and show that Tregs are often present in altered numbers in patients with mood disorders. We hypothesize that Tregs exert their regulatory and protective functions to alleviate the pathological symptoms of *T. gondii* infection when it causes neuroinflammation and brain parenchymal damage.

Tregs, accounting for approximately 5%-10% of the total CD4^+^ T cells (Gao et al., 2021), are crucial to maintain self-tolerance and immune homeostasis. Tregs are known as suppressor T cells, which can regulate the functions of other immune cells like DC, macrophages, CD4^+^ T cells, and B cells (Hong et al., 2013; Mohr et al., 2018). Researchers generally divide Tregs into two subtypes, natural regulatory T cells (nTregs) and induced regulatory T cells (iTregs), which act synergistically to enhance immune tolerance in peripheral blood (Curotto de Lafaille and Lafaille, 2009; Yang et al., 2020). Tregs utilize multiple immunomodulatory mechanisms to limit inflammatory responses and enhance immunosuppressive functions, including contact-dependent mechanisms, secretion of immunomodulatory factors like transforming growth factor (TGF)-β and IL-10, and perturbation of target cell metabolism (Georgiev et al., 2019). In pregnant women with seropositive anti-*T. gondii* antibodies, Tregs expressing T-lymphocyte antigen 4 (CTLA-4) increased, while *T. gondii* infection can also affect the expression of TGF-β and IL-10 (Rezende-Oliveira et al., 2020). Tregs down-regulate CD80 and CD86 on antigen-presenting cells (APCs) *via* CTLA-4, thereby hindering the activation of other T cells by APCs. Additionally, CTLA-4 stimulates IDO expression in DC subsets and then induces the catabolism of TRP to metabolites such as KYNA, which can exert immunosuppressive effects through cytotoxicity (Mellor and Munn, 2004; Miyara and Sakaguchi, 2007). Additionally, the immunomodulatory role of TGF-β and IL-10 is the central in *T. gondii*-induced immunopathogenesis (Butler et al., 2013). Adoptive transfer of Tregs extracted from normal pregnant mice into *T. gondii*-infected pregnant mice was found to improve adverse pregnancy outcomes, which may be related to the upregulation of TGF-β and IL-10 expression by Tregs (Liu et al., 2014). TGF-β has neuroprotective effects and affects memory formation and synaptic plasticity (Qiu et al., 2021), and when *T. gondii* infection induces neuroinflammation, TGF-β suppresses inflammation and reduces neuronal damage (Cekanaviciute et al., 2014). Tregs are involved in the immunopathology of *T. gondii* through surface factors (CTLA-4) and cytokines (TGF-β and IL-10), and similarly, Tregs may regulate *T. gondii*-induced mood disorders through this immune mechanism.

*T. gondii* infection can induce intense brain inflammation (Batista et al., 2020), and the development of mood disorders has been associated with CNS inflammation. Tregs may indirectly affect mood disorders by modulating immune-inflammatory responses. Adolescents at high risk for mood disorders had fewer Tregs than normal healthy individuals, and were often accompanied by symptoms of high inflammation (Snijders et al., 2016). Combining several studies, it has also been found that MDD patients tended to have increased Th1/Th2 and Th17/Tregs ratios when the number of Tregs in peripheral blood decreased, indicating that the decreased number of Tregs may be related to the immune imbalance in MDD patients (Li et al., 2010; Chen et al., 2011). And by investigating the alterations of cytokines and Tregs during antidepressant treatment in 16 MDD patients, it was found that the number of Tregs increased while the production of anti-inflammatory factors decreased during treatment (Himmerich et al., 2010). In animal studies, increased levels of peripheral blood Tregs were also found in mice treated with the antidepressant *Shen-Qi-Jie-Yu-Fang* (SJF) or fluoxetine for postpartum depression (Li et al., 2016). When Tregs-deficient and wildtype mice were simultaneously exposed to stress, the former were more prone to anxiety- and depression-like behaviors, accompanied with increased levels of pro-inflammatory factors (Kim et al., 2012). Xu et al.(Xu et al., 2021b) infected female mice with soluble tachyzoite antigens of *T. gondii* to induce maternal immune activation, and offspring mice developed anxiety-like behaviors. Then, adoptively transferring the pathogen-activated maternal Tregs into the offspring mice, could improve abnormal behavior symptoms of offspring mice.

After *T. gondii* invades the CNS, it can lead to neuronal apoptosis and cause severe damage to the brain parenchyma (An et al., 2018). Many areas of the brain are involved in regulating emotions, including areas such as the frontal cortex, hippocampus, striatum, and amygdala, and they form neural circuits associated with mood disorders (Nestler et al., 2002), while patients with mood disorders have pathological features such as the reduced survival of neuroblasts and immature neurons, damaged neural circuits, and decreased levels of neurotrophic factors (Eyre and Baune, 2012). In Tregs-deficient mouse models, Tregs was found to stimulate oligodendrocyte differentiation and myelin regeneration in the CNS (Dombrowski et al., 2017). During ischemic brain injury, Tregs will accumulate in large numbers in the mouse brain and promote neurological recovery and inhibit neurotoxic astrocyte proliferation by producing amphiregulin (Ito et al., 2019). Furthermore, in a mouse model in which mice were infected with human immunodeficiency virus-1 (HIV-1), Tregs were found to suppress inflammatory cytokines and reactive oxygen species (ROS) production, improve neurotoxicity and increase neuronal survival, while upregulating the expressions of brain-derived neurotrophic factor (BDNF) and glial cell-derived neurotrophic factor (GDNF) (Liu et al., 2009). Tregs can also exert neuroprotective effects by producing TGF-β and IL-10 (Kipnis et al., 2004; Reynolds et al., 2007), which can activate microglia and increase neuronal survival in the hippocampus (Kipnis et al., 2004), and TGF-β can also increase the potency of neurotrophic factors (Rook et al., 2011). Concurrently, in a survey of women with prenatal depression, it was found that TGF-β could regulate MDD during pregnancy, optimize the development of the amygdala in offspring, and reduce the incidence of MDD in offspring (Qiu et al., 2021).

We mentioned above that Tregs can regulate immune homeostasis and suppress inflammation, and have neuroprotective effects that may improve mood disorders. However, *T. gondii* infection often leads to a decrease in Tregs levels, which may exacerbate the symptoms of *T. gondii* -induced mood disorders, suggesting that Tregs and the cytokines TGF-β and IL-10 are important modulators. However, there are no trials that have examined *T. gondii* infection, Tregs and mood disorders together, which is an important direction that could be of great help in both the treatment of *T. gondii* infection and the regulation of mood disorders.

## Th17 cells and mood disorders

Th17 cells, a subtype of CD4^+^ T cells (Harrington et al., 2005), are a unique pro-inflammatory lineage of Th cells that were originally identified and named for the production of the pro-inflammatory factor IL-17 (Miossec and Kolls, 2012; Yang et al., 2014). IL-17 is the major functional cytokine with pro-inflammatory effects expressed by Th17, and only Th17 cells among helper T cells enable to produce IL-17 (Gu et al., 2013; Ghosh et al., 2022). Th17 cells produce a series of inflammatory cytokines like IL-22, IL-21, and IL-26 as well (Yang et al., 2014). Th17 cells play dual functions in the process of *T. gondii* infection. In the early stage of infection, Th17 can resist the invasion and damage of *T. gondii*, while in the later stage, Th17 can trigger immune pathological changes in the body, resulting in severe inflammatory response (Ma et al., 2020). Clinical studies have found that Th17 cells and the proinflammatory factor IL-17 are increased in toxoplasmosis (Dutra et al., 2013; Silva et al., 2014), and IL-17 will further promote the inflammatory response and expand tissue damage after *T. gondii* infection (Guiton et al., 2010). IL-17 also plays a central role in the ocular pathology of *T. gondii*, and it can act as a hallmark inflammatory factor in ocular toxoplasmosis (Greigert et al., 2020). This suggests that Th17 cells are involved in the immune inflammatory response to *T. gondii* and play an essential role in it.

Th17 cells can promote inflammation, attract macrophages and neutrophils to the site of pathology, and release a series of inflammatory products like IL-17, IL-22, IL-21, and cysteine–cysteine motif chemokine ligand 20 (CCL-20) to help amplify the inflammatory response (Dong, 2008). Th17 cells have been shown to be highly pro-inflammatory and can induce severe autoimmunity (Bettelli et al., 2007). Therefore, since Th17 cells were first identified in 2005, many inflammatory diseases and autoimmune diseases have been found to be related to Th17 cells like multiple sclerosis (MS), psoriasis, systemic lupus erythematosus (SLE), etc. (Tesmer et al., 2008). Notably, in a cohort study of patients suffered from SLE and RA based on a large population, up to 50% of patients with autoimmune diseases exhibited depressive-like symptoms (Pryce and Fontana, 2017). As an essential activator of autoimmunity, is Th17 cells associated with the pathological progression of MDD? We were inspired by a study of 40 MDD patients and 30 healthy control subjects, which measured serum antinuclear antibody (ANA) levels, the number of Th17 cells and Tregs in peripheral blood, serum IL-17 concentration, and RORγt expression levels. The results showed that compared with the control group, Th17 cells in peripheral blood from MDD patients were increased, accompanied by the decreased number of Tregs and increased production of IL-17 and RORγt. The positive rate of ANA as an autoimmune indicator in the MDD patients (around 27.50%) was also higher than that in the healthy individuals (around 5.00%), which indicated that Th17 cells were most likely involved in the pathological mechanism of MDD (Chen et al., 2011).

Th17 cells are vital regulators in the pathological development of mood disorders by affecting neuroinflammation (Slyepchenko et al., 2016; Ghosh et al., 2022). It has been shown that Th17 cells can invade the CNS and induce neuroinflammation, thereby mediating the development of schizophrenia (Debnath and Berk, 2014). Th17 cells can also invade the brain parenchyma, release inflammatory cytokines (IL-17 and IL-22) or induce neuroinflammation and neuronal apoptosis through the Fas/FasL pathway to participate in the etiopathogenesis of AD (Zhang et al., 2013a). In the experimental autoimmune encephalomyelitis (EAE) model, Th17 cells were also found to be involved in nerve damage in MS neuroinflammation (Siffrin et al., 2010). And in a mouse model of learned-helplessness, Beurel et al. (Beurel et al., 2018) detected abundant pro-inflammatory Th17 cells in the prefrontal cortex (PFC) and hippocampus, two brain sites whose functions are associated with MDD. Peripheral Th17 cells may cross the BBB into the brain parenchyma under the action of several cytokines, chemokines, and adhesion factors (Sonar and Lal, 2017), such as cysteine–cysteine motif chemokine ligand 20 (CCL20) and chemokine receptor 6 (CCR6), which can induce and assist this process (Zhang et al., 2013b). Th17 cells can activate microglia in the CNS (Murphy et al., 2010; Slyepchenko et al., 2016). Microglia have been found to express IL-17 receptors, and IL-17 can stimulate microglia to produce inflammatory cytokines (e.g., IL-6) by acting on the IL-17 receptor in microglia (Kawanokuchi et al., 2008). This suggests that Th17 cells may induce neuroinflammation by activating microglia. Activated microglia can release pro-inflammatory cytokines to expand neuroinflammation, produce ROS, eicosanoids mediate neurotoxicity, and thus affect neuroplasticity, all of which have been involved in the etiopathogenesis of MDD (McNally et al., 2008). Th17 cells can promote the production of ROS and act on endothelial cells to induce BBB injury, so as to make peripheral immune cells and cytokines enter the brain parenchyma and promote brain inflammation, or IL-17 can directly play a neurotoxic role and lead to brain injury (Huppert et al., 2010; Waisman et al., 2015).

In truth, although many studies have shown a correlation between Th17 cells and mood disorders, the roles and mechanisms of Th17 cells in mood disorders have not been studied extensively, possibly owing to the comparatively late discovery of Th17 cells. Above we summarized that Th17 cells are involved in immune responses during *T. gondii* infection as pro-inflammatory cells and that Th17 cells and IL-17 can promote inflammation in the brain parenchyma. We have reason to believe that Th17 cells are involved in the pathogenesis of mood disorders and that this pathogenesis may be highly relevant to *T. gondii*-induced mood disorders. Therefore, we can next use Th17 cells as an important research target. In addition, the mechanism by which Th17 cells enter the CNS and affect the neuroinflammation of mood disorders remains unclear, and more research is needed to verify. However, there is no doubt that the mechanism of Th17 cells affecting mood disorder *via* affecting neuroinflammation has broad research prospects.

## Th1, Th2 and mood disorders

Th1 cells and Th2 cells are two important Th cell subtypes (Berger, 2000). Th1 cells are associated with cell-mediated inflammation and delayed-type hypersensitivity reactions, mainly secreting cytokines such as IL-2, IFN-γ and TNF. While Th2 is mainly associated with allergic reactions (like asthma) and specific diseases (like MS), it mainly produces IL-10, IL-4 and IL-5 (Raphael et al., 2015). In the immune response, Th1 cells mainly exhibit pro-inflammatory properties, while Th2 cells mainly have anti-inflammatory properties, and the balance of these two cells has a positive effect on maintaining the immune balance of the human body. Mood disorders are associated with immune balance, and some studies have demonstrated Th1 and Th2 cell imbalances in MDD and BD patients (Myint et al., 2005; Brambilla et al., 2014). Significantly, *T. gondii* infection also involves alterations in Th1 and Th2 cells and cytokines (Xu et al., 2021a). In *T. gondii* infection, the body produces a strong Th1 cell response to resist pathogen invasion (Jankovic et al., 2010; Cortez et al., 2014), while Th1 cells also express IL-27 and IFN-γ to influence the development and differentiation of Tregs (Jankovic et al., 2007; Hall et al., 2012).

The imbalance of Th1 and Th2 may be involved in the pathophysiology of MDD. In the study of immune imbalance in MDD patients, it was found that the ratio of Th1/Th2 in peripheral blood of patients was increased (Li et al., 2010), and the decrease of Th2 cells also appeared in patients with MDD (Becking et al., 2018). One study examined the relationship between Th1 and Th2 cytokines and MDD, and then found that patients suffered from MDD had substantially higher levels of TNF-α, which is secreted primarily by Th1 (Huang and Lee, 2007). Another recent study similarly found elevated TNF-α in patients with MDD (Alvarez-Mon et al., 2021). A 2020 analysis of peripheral blood cytokines in 5166 MDD patients and 5083 controls also demonstrated significantly reduced IL-4 levels in MDD patients (Osimo et al., 2020). IL-12 can promote Th1 cell responses, favoring the differentiation of Th1 cells. One study detected high level of IL-12 in the peripheral blood of MDD patients, but IL-12 levels decreased after treatment (Kim et al., 2002). Changes in IL-12 levels during this process may be positively correlated with Th1 cells. In addition, elevated Th1 cell levels were also found in common models of MDD in mice (learned helplessness and chronic restraint stress) (Beurel et al., 2013). These studies indicated that MDD may be linked with a Th1/Th2 imbalance that favors Th1 cells, which may be linked to the pro-inflammatory properties of Th1 cells. Interestingly, behavioral abnormalities and hostility have been suggested to be associated with high level of Th1 cytokine-mediated inflammation (Janicki-Deverts et al., 2010). During *T. gondii* infection, IFN-γ can drive a powerful immune response to inhibit parasite proliferation, and microglia can be activated by it to fight *T. gondii* infection (Lima and Lodoen, 2019; Mukhopadhyay et al., 2020). A review also described the promotion of MDD by the pro-inflammatory factor IFN-γ, which induces IDO1, activates hypothalamic-pituitary-adrenal (HPA) axis as well as microglia, and drives Th1 cell infiltration into the CNS, thus participating in the pathogenesis of MDD (Inserra et al., 2019). Although there is no clear evidence, it can be speculated that Th1 cells are involved in the neuroinflammation of MDD. In parallel, BD patients also have Th1/Th2 imbalance. The Th1 chemokine receptor CCR5 is downregulated in BD, whereas the typical cytokine IL-4 produced by Th2 is upregulated, suggesting an imbalance between Th1 and Th2 cells (Brambilla et al., 2014). Asthma is often comorbid with BD, and one experiment using an integrin β4 (ITGB4)-deficient mouse model to study the association between airway inflammation and BD-like behavior found that the Th2 inflammatory response promotes BD-like behavior in asthmatics (Han et al., 2018).

So far, *T. gondii* infection has been shown to induce a Th1 response, but little research has been done on the relevance and mechanisms of Th1 and Th2 cells to mood disorders. Most of the above related studies only reflect the levels of Th1 cells and Th2 cells by detecting cytokines, which has limitations. Some cytokines (IFN-γ, TNF-α, IL-4, etc.) are not specifically expressed by Th1 cells or Th2 cells, and may also be due to alterations in some living habits (such as smoking, drinking, etc.) (Brambilla et al., 2014). Therefore, further studies need to be conducted, and cells like Th1 and Th2 cells as well as some cytokines (e.g. IFN-γ) may become targets for further research on *T. gondii*-induced mood disorders.

## Microglia and mood disorders

Mood disorder belongs to a neuropsychiatric disease in which microglia plays a vital role (Beumer et al., 2012), which is evidenced by the fact that activation of microglia has been found in both MDD and anxiety disorders (Frick et al., 2013). And one article exposes that MDD can be caused by altered microglia function and therefore can be considered as a microglial disorder (Yirmiya et al., 2015). As resident macrophages in the CNS (Kettenmann et al., 2011), microglia is mainly activated by damage-associated molecular patterns (DAMPs) and pathogen-associated molecular patterns (PAMPs) (Spittau et al., 2020). Those activated microglia can be divided into two phenotypes: the pro-inflammatory/injury-inducing M1 phenotype (M1-type microglia) and the anti-inflammatory/neuroprotective M2 phenotype (M2-type microglia) (Tschoe et al., 2020). M1-type microglia can induce hippocampal neuronal apoptosis by producing inflammatory cytokines like IL-6 (Monje et al., 2003), IL-1β (Goshen et al., 2008), and TNF-α (Cacci et al., 2005), inhibit neurogenesis, and produce NO and ROS for neurotoxicity, while the intracellular IDO enzyme is activated to promote the production of KYNA and QUIN by the tryptophan pathway as well (Jia et al., 2021). M2-type microglia have anti-inflammatory effects, promote inflammation regression, repair damage, and release nutritional factors (Zhao et al., 2015). Taken together, these functional changes after microglia activation can affect the occurrence and development of MDD.

Within *T. gondii* infection, *T. gondii* can enter the brain parenchyma and form brain cysts, causing dopamine dysregulation. Tachyzoites can infect about 30% of microglia and cause CNS inflammation. In addition, after infection with *T. gondii*, CD4^+^ T cells and CD8^+^ T cells secrete inflammatory factors such as TNF-α and IL-1 to stimulate microglia, and interfere with gamma-aminobutyric acid (GABA) and 5-hydroxytryptamine (5-HT) synthesis *via* the KYNA pathway (Mukhopadhyay et al., 2020; Yin et al., 2022). This may be the physiological basis as well as the contributing factor for *T. gondii*-induced mood disorders and behavioral abnormalities.

In MDD, microglia are primarily activated by immune cells or inflammatory cytokines in infectious or non-infectious inflammatory responses (Yirmiya et al., 2015). CD4^+^ T cells can cross the BBB, infiltrate the CNS, directly or indirectly interact with microglia, and participate in the activation of M1 or M2 microglia (Solleiro-Villavicencio and Rivas-Arancibia, 2018; Das and Chinnathambi, 2019). Among them, Th1 and Th17 cells contribute to M1-type cell transformation and promote neuroinflammation, while Tregs and Th2 induce microglia anti-inflammatory M2 phenotype and neuroprotection (Gonzalez and Pacheco, 2014). M1 polarization is dependent on IFN-γ secreted by Th1 cells and IL-17 expressed by Th17 cells. Similarly, IL-2 and IL-10 produced by Th2 cells and TGF-β and IL-10 produced by Tregs can promote M2 polarization of microglia (Chen et al., 2020; Tschoe et al., 2020). Bias of Th1/Th2 or Th17/Tregs can de-affect the development of MDD by affecting microglial M1/M2 polarization, while the role of inflammation and inflammatory factors runs through it, whether it is possible to try to push microglia to the M2 phenotype, improve neuroinflammation and protect nerves, and then achieve the purpose of treating MDD.

During *T. gondii* infection of the CNS, microglia can respond to *T. gondii* infection by responding to and releasing pro-inflammatory factors and recruit T cells into the brain to fight the infection by releasing chemokines CXCL9 and CXCL10. Microglia are also specialized APCs in the CNS, and some microglia (~3%) can express MHC class II molecules to help activate CD4^+^ T cells to maintain immune defenses (Cowan et al., 2022). On the other hand, the inflammatory response due to microglia activation may lead to neurodegeneration and neurotoxicity, and this immune activation is detrimental. Therefore, during *T. gondii* infection of CNS, an immune balance between control of the pathogen and immune tolerance should be emphasized, and excessive *Toxoplasma* virulence, immunodeficiency and overly strong immune responses can compromise this balance and lead to CNS pathology or death. Next, we should further investigate the anti-infective function of microglia and their neuroprotective role in psychiatric disorders such as mood disorders.

## Summary

A growing body of data support the idea that *T. gondii* infection promotes mood disorders. *T. gondii* is a widespread and complex parasite that infects humans universally and establishes lifelong infection, but causes widely varying clinical symptoms. In immunocompetent individuals, lifelong infection with *T. gondii* does not cause symptoms, but in immunocompromised individuals such as AIDS patients and fetuses, *T. gondii* infection inducing a variety of toxoplasmosis diseases greatly increases the risk of death. Therefore, the outcome of the infection depends to a large extent on the immune capacity of the host. In addition, different types of strains cause very different consequences. Compared to type II and type III strains, type I strains tend to be rigidly virulent and can lead to easy death of the host, while type II strains can induce a stronger inflammatory response, causing inflammatory diseases such as encephalitis or ileitis. It is worth noting that most of the past studies on *T. gondii*-induced behavioral changes used different types of *T. gondii* strains (e.g., RH, HIF, PRU, etc.), and this may lead to different experimental results due to *T. gondii* strain variability. Type I strains are too virulent and often lethal to the host, while type II and III strains are relatively less virulent, which may make it easier to establish chronic infections in the host, while facilitating our study of behavioral changes due to chronic infections. Therefore, the choice of strains to study psychiatric diseases in the future is a question worth considering. Moreover, the susceptibility of different hosts to *T. gondii* varies, with different genetic backgrounds and immune mechanisms involved. Most of the experimental animal models we discussed above are primarily of mouse origin, so we should consider the question: can *T. gondii*-induced behavioral changes in animals really be analogous to humans? Some simple behavioral changes may be possible, and one article gives us empirical evidence to support this analogy (Flegr, 2013). However, when it comes to psychiatric disorders with more complex symptoms and mechanisms, such as MDD, bipolar disorder, and schizophrenia, we should consider whether the complexity of these disorders can be reflected by animal models. But there is no doubt that animal models will help us to understand all aspects of psychiatric disorders, providing us with novel insights in the epidemiology and pathophysiology of *T. gondii*-induced mood disorders.

In recent years, research on the association between *T. gondii* and mood disorders has gradually increased, with a growing number of epidemiological studies highlighting the potential role of *T. gondii* in the pathogenesis of mood disorders, and above we show the correlation between them. However, this correlation may be bidirectional, as individuals with psychiatric disorders may be more susceptible to exposure and increased susceptibility to neurotropic pathogens due to behavioral abnormalities or reduced immune competence. Infection with neurotropic pathogens such as *T. gondii* can lead to neuronal damage, central inflammation, neurotransmitter and hormonal changes that can cause and exacerbate behavioral changes or neuropsychiatric disorders. Most current models of *T. gondii*-induced mood disorders suggest that changes in brain parenchyma and altered immune status lead to cognitive and emotional deficits and manifest as mood disorder symptoms such as behavioral abnormalities. However, it is noteworthy that most of the current studies on the role of *T. gondii* in mood disorders are associative and do not establish a causal relationship, and the exact mechanism of their influence remains unknown. Here we summarize the association between *T. gondii*, the immune system and mood disorders, and attempt to build a framework for the “immune-brain axis” of *T. gondii*. We also show evidence that inflammation is an intermediate pathway between *T. gondii* and mood disorders, and that *T. gondii* triggers an inflammatory process that leads to structural and functional damage in the CNS, causing changes in mood, social behavior, and cognitive performance. In this review, we explore the ability of *T. gondii* to cause alterations in CD4^+^ T cell numbers and function and the link between several important CD4^+^ T cells and mood disorders, including Tregs, Th1, Th2, and Th17 cells. These cells and secreted inflammatory factors are involved in immune inflammatory responses and can be recruited to the CNS, activate microglia, and participate in processes such as neuroinflammation and neurotoxicity. We therefore propose a reasonable hypothesis that inflammation has a positive effect on mood disorders and that CD4^+^ T cells may be involved in the pathogenesis of *T. gondii* -induced mood disorders by regulating and participating in immune inflammatory mechanisms. The involvement of *T. gondii* in the pathology of mood disorders through immunoinflammatory mechanisms may be an attractive theoretical framework. Of course, this framework currently lacks experimental studies on the causal relationship between *T. gondii* and mood disorders, so a lot of attempts need to be invested to defeat this series of diseases.

Furthermore, in future studies we should consider whether different strains of *T. gondii* induce different types and degrees of psychiatric disorders in humans? Are patients with Psychiatric Disorders more susceptible to *T. gondii* infection due to changes in their immune system or lifestyle habits? What influences the differences in pathogenicity of *T. gondii* in different intermediate hosts? Can a clear pathological mechanism be established between *T. gondii* and mood disorders through immune cells and inflammatory factors, and do CD4^+^ T cells and associated inflammatory factors represent new and effective therapeutic targets for mood disorders, especially those caused by *T. gondii* infection? In conclusion, more in-depth studies are needed to explore the mechanisms linking *T. gondii* and mood disorders., **2**

## Author contributions

Conception or Design of the Work: JC; Drafting the Article: QW, YZ, NC, and JC; All authors reviewed the manuscript. All authors contributed to the article and approved the submitted version.
